# Radiomic assessment as a method for predicting tumor mutation burden (TMB) of bladder cancer patients: a feasibility study

**DOI:** 10.1186/s12885-021-08569-y

**Published:** 2021-07-16

**Authors:** Xin Tang, Wen-lei Qian, Wei-feng Yan, Tong Pang, You-ling Gong, Zhi-gang Yang

**Affiliations:** 1 Department of Radiology, West China Hospital, Sichuan University, 37# Guo Xue Xiang, Chengdu, 610041 Sichuan China; 2 Department of Thoracic Oncology and State Key Laboratory of Biotherapy, Cancer Center, West China Hospital, Sichuan University, 37# Guo Xue Xiang, Chengdu, 610041 Sichuan China

**Keywords:** Bladder cancer, Radiomics, Tumor mutation burden, Driver mutations, Predictive model

## Abstract

**Background:**

Tumor mutation burden (TMB) is an emerging prognostic biomarker of immunotherapy for bladder cancer (BLCA). We aim at investigating radiomic features’ value in predicting the TMB status of BLCA patients.

**Methods:**

Totally, 75 patients with BLCA were enrolled. Radiomic features extracted from the volume of interest of preoperative pelvic contrast-enhanced computed tomography (CECT) were obtained for each case. Unsupervised hierarchical clustering analysis was performed based on radiomic features. Sequential univariate Logistic regression, the least absolute shrinkage and selection operator (LASSO) regression and the backward stepwise regression were used to develop a TMB-predicting model using radiomic features.

**Results:**

The unsupervised clustering analysis divided the total cohort into two groups, i.e., group A (32.0%) and B (68.0%). Patients in group A had a significantly larger proportion of having high TMB against those in group B (66.7% vs. 41.2%, *p* = 0.039), indicating the intrinsic ability of radiomic features in TMB-predicting. In univariate analysis, 27 radiomic features could predict TMB. Based on six radiomic features selected by logistic and LASSO regression, a TMB-predicting model was built and visualized by nomogram. The area under the ROC curve of the model reached 0.853. Besides, the calibration curve and the decision curve also revealed the good performance of the model.

**Conclusions:**

Our work firstly proved the feasibility of using radiomics to predict TMB for patients with BLCA. The predictive model based on radiomic features from pelvic CECT has a promising ability to predict TMB. Future study with a larger cohort is needed to verify our findings.

**Supplementary Information:**

The online version contains supplementary material available at 10.1186/s12885-021-08569-y.

## Background

The past several years have witnessed the blooming of immune checkpoint inhibitors (ICIs) targeting the programmed death-1 (PD-1) pathway in cancer treatment [1]. As the most common tumor of the urinary system, bladder cancer (BLCA) is recognized as one of the immunologically “hot” tumors [2], and thereby, a good candidate for immunotherapy. Till now, the FDA has approved three programmed death-L1 (PD-L1) inhibitors, i.e. atezolizumab, durvalumab, and avelumab, as well as two PD-1 inhibitors i.e. nivolumab and pembrolizumab in the treatment of urothelial carcinoma [3].

Although the emergence of immunotherapy brings hope for patients of BLCA, it cannot be ignored that this novel treatment is not always effective in all patients. Besides, immunotherapy can also cause toxic and potentially fatal side effects [4]. Therefore, identifying biomarkers that can distinguish the potential responders of ICIs from the non-responders is essential for accurate treatment decisions. PD-L1 expression and tumor mutation burden (TMB) are the two most commonly used biomarkers [5, 6]. Unlike the detection of PD-L1 expression that focuses on the targeting protein of the ICIs, TMB predicts the therapeutic efficacy of ICIs through its strong correlation to the mutation-derived neoantigens which is a key factor for immune response activation [5]. Studies even showed that TMB is superior to PD-L1 in predicting the therapeutic efficacy of ICIs therapy [7, 8]. Besides, it has been reported that higher TMB is associated with a favorable prognosis of ICIs treatment in tumors including melanoma, non-small-cell lung cancer, small-cell lung cancer, urothelial cancer [9–13] and etc. However, a major obstacle that prevents the large-scale promotion of TMB detection in patients receiving ICIs is the high cost of the whole-exome sequencing (WES) test.

Radiomics is a rapidly emerging field that can be applied to many biomedical areas [14]. Studies have shown that radiomic features are capable of predicting the somatic mutation of certain genes in different tumor types [15–17]. Moreover, two latest studies addressed that radiomics could also predict the TMB status of lung cancer patients [18, 19]. These studies revealed the deep connection between the radiomic and genomic characteristics in cancer patients and the feasibility of using radiomic features to predict the genomic outcomes. Therefore, we hypotheses that radiomic features can be used to select the most clinically needed patient population for TMB testing by predicting the probability of high TMB. Besides, radiomics may act as an alternative or assistant diagnosis of TMB detection for those who are not accessible to the expensive WES test.

In this study, we aim at exploring the value of radiomic features extracted from pelvic contrast-enhanced computed tomography (CECT) images in predicting the TMB status of BLCA patients, and also, developing a TMB-predicting model based on the radiomic data.

## Methods

### Study population and data acquisition

**Fig. 1:**
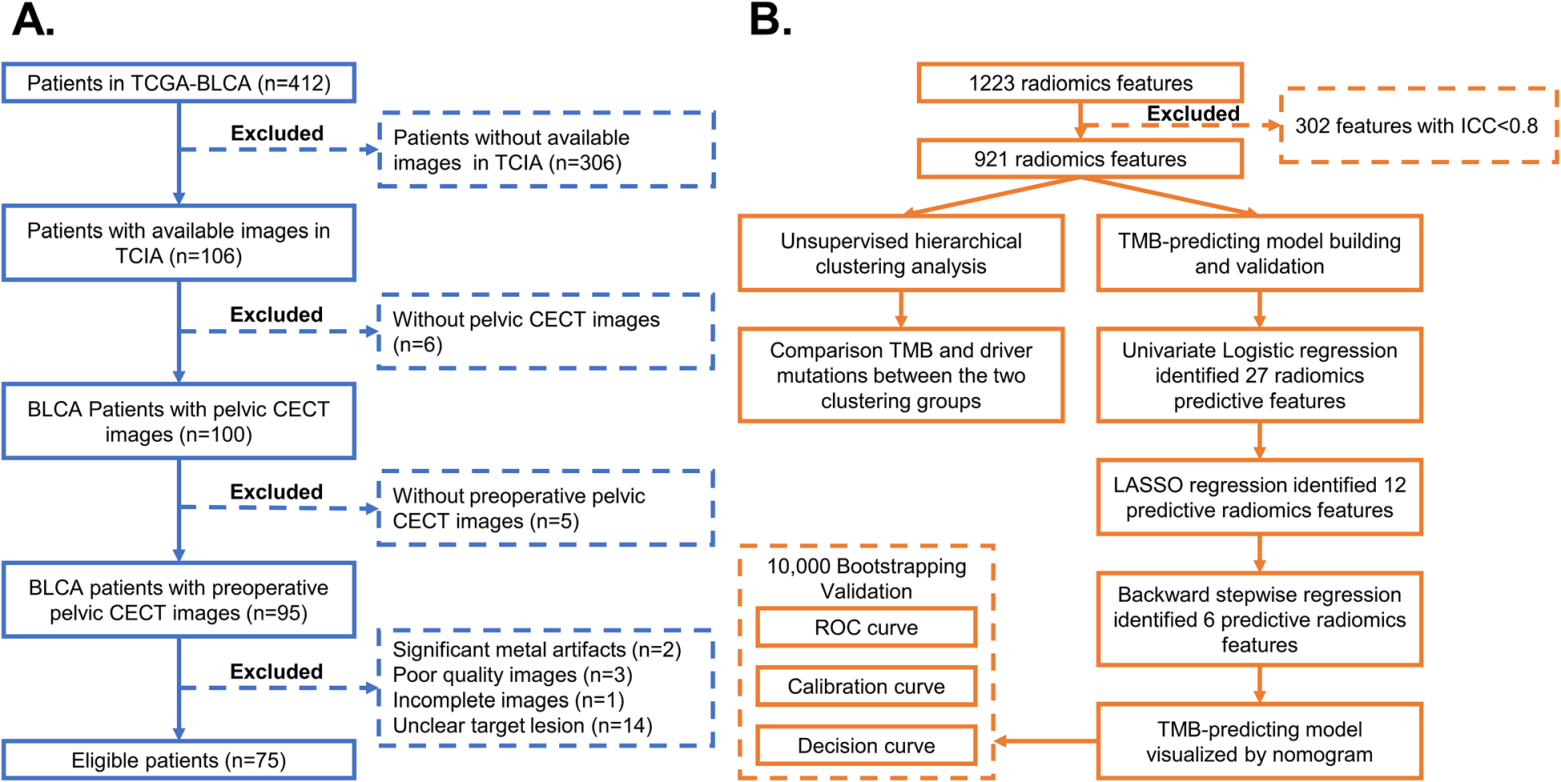
Flowchart showing the inclusion criteria (**A**) and the detailed analytic processes (**B**) of the current study. TCGA: The Cancer Genome Atlas; TCIA: The Cancer Imaging Archive; BLCA: Bladder cancer; TMB: Tumor mutation burden; CECT: Contrast-enhanced computed tomography; LASSO: Least absolute shrinkage and selection operator; ROC: Receiver operating characteristic; ICC: interclass correlation coefficient

Pelvic CECT images of eligible BLCA patients were downloaded from the Cancer Imaging Archive database (TCIA, http://www.cancerimagingarchive.net/) [20]. Genetic and clinical data were acquired from the Cancer Genome Atlas (TCGA) database (http://cancergenome.nih.gov) [21]. The Inclusion criteria are shown in Fig. 1A, including: 1) pathological diagnosis as BLCA; 2) available preoperative pelvic CECT images with good quality; 3) available genetic information. Finally, 75 eligible BLCA patients from the TCGA-BLCA cohort were enrolled. No ethical approval nor informed consent was required for the current study due to the public availability of data in the TCIA and TCGA databases.

### CT imaging parameters

The preoperative pelvic CECT images were obtained from four various manufactures: General Electric (GE), Siemens, Philips and Toshiba Medical Systems. The acquisition parameters of CT were as follows: slice thickness, 1.25 - 5 mm; tube voltage, 100–140 kV; tube current, 80–689 mA; matrix, 512 × 512; and pixel size, 0.586 × 0.586 mm^2^ to 0.977 × 0.977 mm^2^.

### The volume of interest (VOI) delineation and feature extraction

All pelvic CECT images were obtained before surgery. On the axial CECT image, two radiologists used the open-source software 3D slicer (Version 4.10.2) [22] to manually delineate the VOI of tumor independently.

Imaging pre-processing steps were conducted to decrease the potential protocol variability. Prior to feature extraction, all CT images were resampled into voxel sizes 1 × 1 × 1 mm^3^ and discretized to a bin width of 25 Hounsfield units (HU). Finally, 1223 radiomic features including shape, first order and texture features with and without performing Wavelet and Laplacian of Gaussian (LoG) filter were acquired. Texture features were classified into five categories including gray-level co-occurrence matrix (GLCM), gray-level difference matrix (GLDM), gray-level run length matrix (GLRLM), gray-level size zone (GLSZM) and neighborhood gray tone difference Matrix (NGTDM).

Combat algorithm was applied for feature harmonization in different imaging protocols from multicenter investigations (https://github.com/Jfortin1/ComBatHarmonization) [23, 24]. All the radiomic features were normalized by the Z-score transformation. The consistency of radiomic features between the two radiologists was assessed by interclass correlation coefficient (ICC). Only stable features with ICC > 0.8 were included in the further analysis (Fig. 1B).

### Tumor mutation burden

In this study, TMB calculation was based on somatic nonsynonymous mutation, while synonymous mutation was excluded. For each patient, TMB was counted as the total mutational count divided by the exome size (estimated as 38 Mb) [25]. Based on the median TMB of all patients, the total cohort was divided into high and low TMB groups. Besides, the top 20 driver mutations of BLCA (obtained from the driver mutation database IntOGen [26]) were also included in the analyses.

### Unsupervised hierarchical clustering analysis

Unsupervised hierarchical clustering analysis was performed to identify distinct subgroups of BLCA patients based on the homogeneity and heterogeneity of the radiomic features. Genomic outcomes and clinical data were compared between the clustering groups by chi-square test. Based on the similarity of radiomics among samples calculated by Euclidean distance, hierarchical clustering can split the total cohort into different subgroups with high radiomic similarity within each subgroup while distinct radiomic profile between subgroups. Unsupervised hierarchical clustering analysis was conducted using the “pheatmap” R package.

### Development and validation of the TMB-predicting model

The detailed produces of radiomic TMB-predicting model building were described as follow: Firstly, univariate logistic regression analysis was used to preliminarily screen and identify potential TMB-predictors from radiomic features. Then radiomic features with *p* < 0.05 in univariate analysis were further examined by the least absolute shrinkage and selection operator (LASSO) regression methods via 10-fold cross-validation based on minimum criteria. In addition, multivariate logistic regression using backward elimination strategy was performed to eliminate the redundant features. Finally, TMB-predicting model based on simplified radiomic features was established.

The novel TMB-predicting radiomic model was visualized as nomogram. Besides, the performance of this predictive model was evaluated using 10,000 bootstrapping method. The area under the receiver operating characteristic (ROC) curve (AUC), calibration curve and decision curve were used to assess the discrimination ability, calibration and clinical benefit of the model, respectively.

### Statistical analyses

Radiomic features were extracted from pelvic CECT using the 3D slicer software. Statistical analyses were conducted by R software (V 3.6.2). All tests were two-sided. A *p*-value < 0.05 was defined as significant for all the tests except that in multivariate logistic regression with backward elimination strategy a *p*-value < 0.1 was considered as significant so that potential predictors were less likely to be eliminated from the predictive model.

## Results

### Baseline characteristics

In total, 75 eligible patients were included in this study according to our inclusion criteria (Fig. 1A). The median TMB of all patients was 6.5 mut/Mb (interquartile range: 3.3–12.1). All patients were divided by the median TMB into the high TMB (49.3%, 37/75) and low TMB (50.7%, 38/75) group. The baseline factors were comparable between those with high and low TMB, except that all the seven black patients in this study harbored low TMB (Table 1). The median follow-up time calculated by the reverse Kaplan-Meier method was 29.5 Months. Totally, death and disease progression occurred in 45.3% (34/75) and 60.0% (45/75) patients, respectively. The median OS and DFS was 35.4 months (95%CI: 21.6–49.1 months) and 25.0 months (95%CI: 12.6–37.3 months), respectively. Log-rank test showed a trend of shorter median OS (35.0 months vs. 56.4 months, *p* = 0.193) in the low TMB group, although the *p*-value were not significant (Fig. S1 A, B).

**Table 1:** Baseline factors of the total cohort and groups with high and low TMB

	Total ***N*** = 75	Low TMB (***N*** = 38)	High TMB (***N*** = 37)	***P*** value
**Age (Y)**				
**< 69**	38 (50.7%)	20 (52.6%)	18 (48.6%)	0.730
**≥ 69**	37 (49.3%)	18 (47.4%)	19 (51.4%)	
**Gender**				
**Male**	57 (76.0%)	28 (73.7%)	29 (78.4%)	0.634
**Female**	18 (24.0%)	10 (26.3%)	8 (21.6%)	
**Race**				
**White**	67 (89.3%)	31 (81.6%)	36 (97.3%)	0.015
**Black**	7 (9.3%)	7 (18.4%)	0 (0.0%)	
**Asian**	1 (1.3%)	0 (0.0%)	1 (2.7%)	
**Diagnosis Year**				
**2005–2010**	30 (40.0%)	16 (42.1%)	14 (37.8%)	0.706
**2011–2013**	45 (60.0%)	22 (57.9%)	23 (62.2%)	
**BMI (kg/m2)**				
**< 26.6**	34 (45.3%)	17 (44.7%)	17 (45.9%)	0.906
**≥ 26.6**	35 (46.7%)	18 (47.4%)	17 (45.9%)	
**Unknown**	6 (8.0%)	3 (7.9%)	3 (8.1%)	
**p T stage**				
**pT2**	24 (32.0%)	11 (28.9%)	13 (35.1%)	0.676
**pT3–4**	43 (57.3%)	22 (57.9%)	21 (56.8%)	
**Unknown**	8 (10.7%)	5 (13.2%)	3 (8.1%)	
**p N stage**				
**pN0**	42 (56.0%)	19 (50.0%)	23 (62.2%)	0.373
**pN1–2**	21 (28.0%)	12 (31.6%)	9 (24.3%)	
**Unknown**	12 (16.0%)	7 (18.4%)	5 (13.5%)	
**Stage**				
**Stage II**	28 (37.3%)	12 (31.6%)	16 (43.2%)	0.296
**Stage III**	47 (62.7%)	26 (68.4%)	21 (56.8%)	
**Clustering Group**				
**Group A**	24 (32.0%)	8 (21.1%)	16 (43.2%)	0.039
**Group B**	51 (68.0%)	30 (78.9%)	21 (56.8%)	

*TMB* Tumor mutation burden; *BMI* Body mass index

### Unsupervised hierarchical clustering analysis

**Fig. 2:**
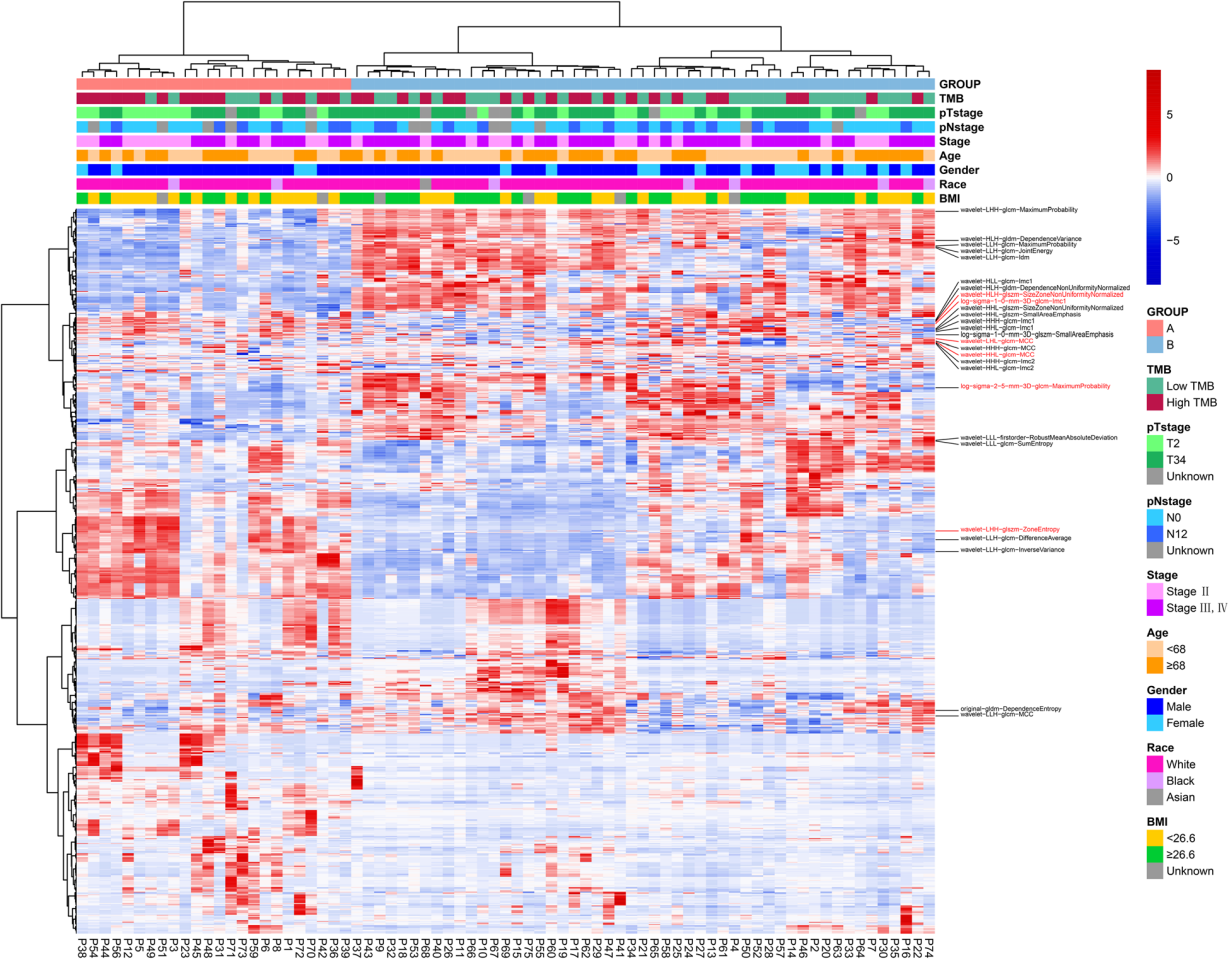
Unsupervised hierarchical clustering analysis of radiomic features. Radiomic features with predictive ability in the univariate analysis are labeled. Radiomic features included in the final TMB-predicting model are marked in red. Based on the homogeneity and heterogeneity of the radiomic features, all cases were divided into two clustering groups by the unsupervised hierarchical clustering analysis. Clinical and genomic outcomes were compared between the two groups. TMB: Tumor mutation burden; BMI: body mass index

**Fig. 3:**
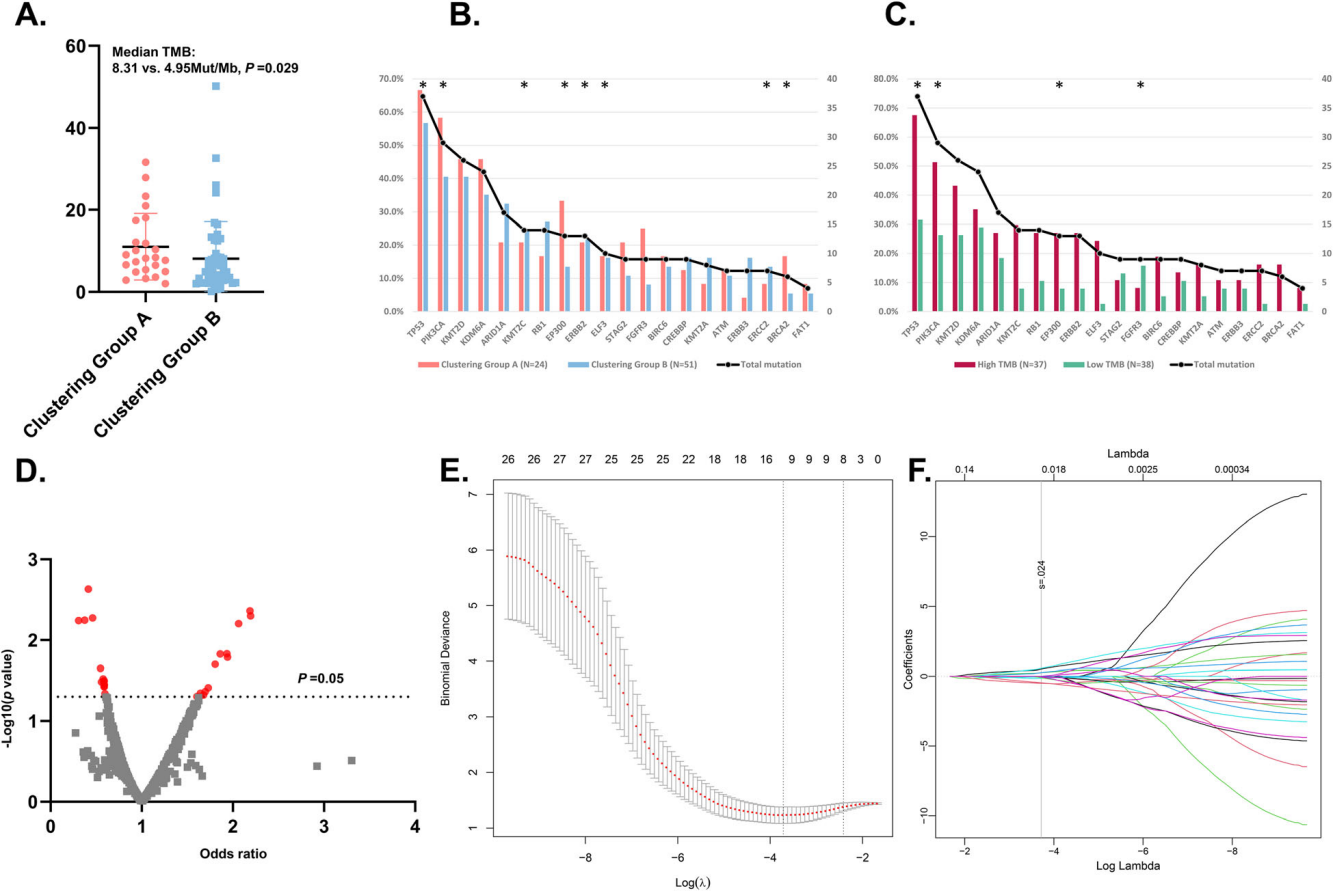
The association between radiomic features and TMB status. **A.** The comparison of TMB as a continuous factor between patients in the clustering group A and B; **B**, **C**. The relationship between the top 20 driver mutations of BLCA **(B)** and radiomic clustering groups or TMB status (**C**), * *p* value < 0.05. **D**. Volcano plot illustrating the results of univariate logistic regression of radiomic features. **E**, **F**. LASSO regression for TMB-prediction based on radiomic features. **E**: The dotted vertical line was plotted at the value selected by the 10-fold cross-validation based on the minimum criteria (the value of lambda with the lowest partial likelihood deviance). **F**: Selection of the tuning parameter (lambda) in the LASSO regression via 10-fold cross-validation based on minimum criteria. TMB: Tumor mutation burden; BLCA: Bladder cancer; LASSO: Least absolute shrinkage and selection operator

The detailed analysis processes of this study are shown in Fig. 1B. To explore the association between radiomic features and clinical outcomes, we performed an unsupervised hierarchical clustering analysis using the 1223 radiomic features with ICC > 0.8. As is shown in Fig. 2, the unsupervised clustering divided the whole cohort into two clustering groups (clustering group A: 24/75 [32.0%]; and clustering group B: 51/75 [68.0%]) with high radiomic similarity within each group while distinct radiomic profile between groups. The clinical factors were compared between the two groups (Table S1). Of note, compared to cases in the clustering group B, those in group A had a significantly higher proportion of having high TMB (66.7% [16/24] vs. 41.2% [21/51], *p* = 0.039) (Table S1). In addition, when taking TMB as a continuous variable, the median TMB was also higher in the clustering group A against group B (8.31 mut/Mb vs. 4.95 mut/Mb, *p* = 0.029, Fig. 3A). Based on the unsupervised nature of the clustering analysis, these findings indicated that radiomic features extracted from CECT have an intrinsic ability in discriminating the TMB status of BLCA patients. Besides, lower T stage and clinical stage were more frequently found in clustering group A than group B (Table S1). In terms of survival outcomes, patients in clustering group A had more favorable OS against those in group B with a borderline *p* value (median OS: not reached vs. 33.0, *P* = 0.058 Fig. S1. C), while the DFS was also numerically longer in group A (35.7 vs. 19.8, *P* = 0.257 Fig. S1. D).

We also explored the relationship between the radiomic features or TMB status with the occurrence of the driver gene mutation. Somatic mutations in genes including TP53, PIK3CA, EP300 and FGFR3 were more frequent in the clustering group A against group B (Fig. 3B). Besides, high TMB was accompanied by more frequent mutations in TP53, PIK3CA, KMT2C, EP300, ERBB2, ELF3, ERCC2 and BRCA2 gene (Fig. 3C).

### Feature selection, model establishment and evaluation

**Fig. 4:**
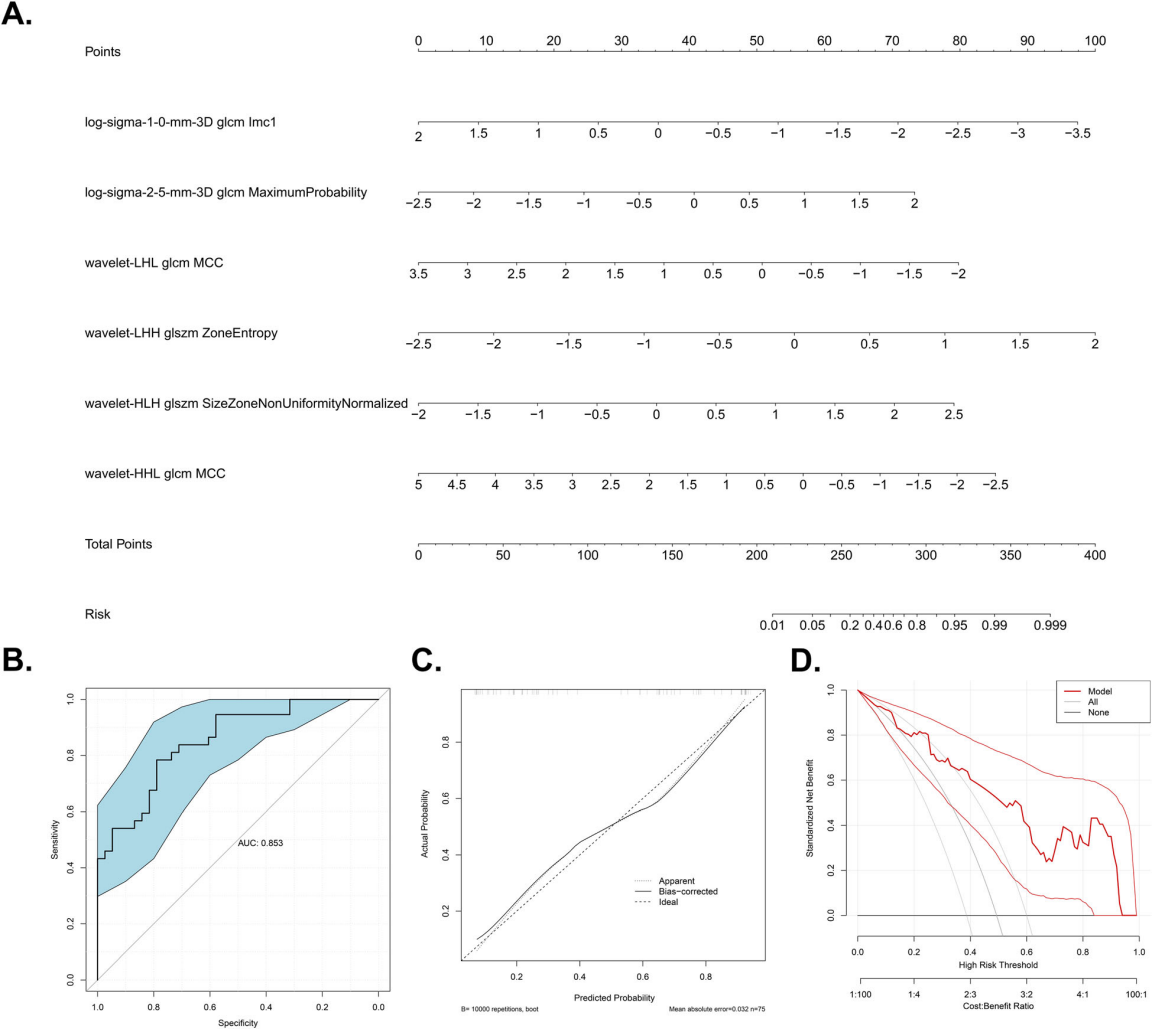
Model visualization using nomogram (**A**) and 10,000 bootstrapping validation (**B-D**). **A**. TMB-predicting model visualized by Nomogram. **B**. ROC curve reflecting the predictive accuracy of the model. Blue area shows the 95%CI of the AUC. **C**. Calibration curves showing the predicted versus actual probability of high TMB status; **D**. Decision curve of the model. The X-axis shows the threshold probabilities while the Y-axis shows the net benefit (adding true positives and subtracting false positives). TMB: Tumor mutation burden; ROC: Receiver operating characteristic

Since clustering analysis showed the strong potential of radiomic features in predicting TMB, we further tested each feature’s power in predicting TMB and developed a TMB-predicting model based on radiomic features. Three sequential steps were involved in the development of the predictive model (Fig. 1B). Firstly, univariate logistic regression analysis was carried out in each radiomic feature. A total of 27 radiomic features with the ability in predicting TMB (*p* < 0.05) were preliminarily identified (Fig. 3D). Then, LASSO regression was conducted using these 27 features to further screen the most powerful prognostic features. 12 radiomic features remained after the LASSO regression (Fig. 3E, F). Afterward, in order to further eliminate the redundant features, these 12 TMB-predicting features were subjected to a backward stepwise logistic regression. Eventually, 6 robust radiomic features were found to be independent predictors of TMB, of which three and three features were positively and negatively related to high TMB status, respectively (Table 2). Finally, the TMB-predicting model was build based on the 6 radiomic features selected by the backward stepwise regression. Based on the beta value of features included in the backward stepwise regression, a TMB-predicting model of radiomic features was established and visualized as a nomogram (Fig. 4A).

**Table 2:** Multivariate Logistic regression using backward elimination strategy

Radiomic Features			Beta value	OR	95%CI OR	***P*** value	AUC^**a**^
log-sigma-1-0-mm-3D	glcm	Imc1	−1.24	0.29	0.10–0.82	0.019	0.637
log-sigma-2-5-mm-3D	glcm	MaximumProbability	1.14	3.13	1.34–7.33	0.009	0.638
wavelet-LHL	glcm	MCC	−1.02	0.36	0.16–0.82	0.015	0.690
wavelet-LHH	glszm	ZoneEntropy	1.56	4.74	1.70–13.20	0.003	0.661
wavelet-HLH	glszm	SizeZoneNonUniformityNormalized	1.23	3.43	1.45–8.13	0.005	0.696
wavelet-HHL	glcm	MCC	−0.80	0.45	0.18–1.12	0.086	0.644

***OR*** **Odds ratio,** ***CI*** **confidence interval**

^**a**^ **Area under the receiver operator characteristic curve**

Correlations within distinct radiomic features were also explored. As is shown in Fig. S2A, correlations were identified among several radiomic features with predictive ability in univariate analysis. LASSO regression is widely used to eliminate multi-collinearity. After feature selection by LASSO regression and backward elimination regression, correlations within radiomic features were dramatically reduced (Fig. S2B, C).

The predictive performance of the TMB-predicting model was validated using 10,000 bootstrapping replications in three distinct aspects as follows. The AUC of the ROC curve was 0.853 (95%CI: 0.770–0.936), reflecting the satisfactory discriminating ability of the model (Fig. 4B). The importance of each radiomic feature in the TMB-predicting model is shown by AUC of ROC curve (Table 2). The highest AUC of ROC curve for a single feature is 0.696. Obviously, the TMB-predicting model harbored much higher predictive accuracy against any radiomic feature alone. Besides, the calibration curve also exhibited good agreement between prediction and observation probability of high TMB (Fig. 4C). In addition, the decision curve analysis demonstrated great positive net benefits among most of the threshold probabilities, indicating the favorable clinical effectiveness of this TMB-predicting model (Fig. 4D).

## Discussion

TMB is a widely used therapeutic biomarker for ICIs treatment in many cancers including BLCA [1]. To the best of our knowledge, this study is the first one to investigate the ability of radiomic features extracted from pelvic CECT images to predict the genomic outcomes of BLCA patients. Our findings revealed an intrinsic connection between radiomic features and TMB status as well as several critical driver mutations. In addition, we initiatively developed a radiomic TMB-predicting model that can be used to predict the TMB status of BLCA patients. Though future validation is still needed, our study reveals the practicability of assessing the TMB status by radiomic features for patients with BLCA.

Radiomics study has developed rapidly in a wide range of fields in oncologic researches for its advantages in capturing comprehensive image information [14]. In BLCA, radiomics exhibited promising potential in predicting pathological grade [27], clinical stage [28], lymph node metastasis [29], recurrence [30], progression-free interval [31] and etc. Here, we firstly found that correlations also existed between radiomic features and genomic alterations. Similar findings were observed in patients with lung cancer [17, 19]. These results implied the possibility that the patient’s genetic changes could be reflected on radiological images and quantified by radiomic features.

In our study, the TMB-predicting model was visualized by nomogram which could conveniently calculate the possibility of high TMB for BLCA patients. This tool has the potential in facilitating clinicians to choose the optimal candidates for TMB testing, i.e., patients that are more likely to harbor high TMB. On the other hand, our model could also serve as an alternative of TMB for patients who cannot afford to the expensive TMB testing or does not have accessible tissue sample for the test. Compared to the traditional TMB detection system, our radiomic TMB-predicting model is totally non-invasive. Furthermore, since most BLCA patients have already undergone the pelvic CECT scan before or at initial diagnosis, this TMB-predicting model requires almost no additional examination for TMB-testing.

BLCA is a highly immunogenic tumor type due to its high mutational load, and consequently, a promising candidate for immunotherapy. At present, a total of 5 ICIs has been approved to be used as either first-line (atezolizumab and pembrolizumab) or second-line (atezolizumab, durvalumab, avelumab, nivolumab and pembrolizumab) treatment schemes for locally advanced or metastatic BLCA [3]. Given the high cost and nonnegligible toxicity of ICIs, identifying biomarkers that can precisely determine the treatment outcomes of ICIs is of great necessity and importance. As one of the targeting molecules of ICIs, PD-L1 expression is recognized as an ideal marker for therapeutic efficacy prediction [6]. Yet, there are also studies addressing that ICIs can be effective in tumors lacking PD-L1 expression [3, 12]. Apart from PD-L1, TMB is another promising biomarker. It is well known that the primary targets of human anti-tumor immune responses are tumor-specific neoantigen peptides originated from somatic mutations in tumors [5]. Accordingly, TMB, which represents the total count of nonsynonymous somatic mutations across the tumor genome, can reflect the antigenicity of tumors. According to a recent meta-analysis, the positive correlations between TMB and ORR in ICIs treatment were found in 27 tumor types [32].

Despite the promising predictive value of TMB in various cancers, TMB detection is now faced with several challenges. One major obstacle for universal TMB testing before ICIs treatment is the high economic cost of the WES examination, which is the golden standard approach for TMB quantifying. Though several relatively cheaper panel-based testing methods have been developed [33, 34], the critical validation of these tools is still lacking. Another annoying aspect of regular WES tests is that it requires an accessible tissue sample which can be hard or even impossible to obtain in some cases. The TMB-predicting model that we established in the current study just makes up for the shortcomings of the above-mentioned traditional TMB detection methods.

This study has several limitations. Firstly, this is a retrospective study with a relatively small sample size, therefore shortcomings connected to its retrospective nature are inevitable. Secondly, since there’s currently no consensus about the optimal cut-off value of TMB in BLCA, we chose to use the median TMB to define the high and low TMB status, which could possibly be varied across different centers. Thirdly, the biological explanation behind radiomics’ ability to predict TMB in patients with BLCA is not yet known. Future study is still needed to clarify this issue. Finally, although a bootstrapping method was used for the model testing, external validation of the model using data from other centers is still needed.

## Conclusion

In this study, we firstly explored the association between radiomic features and TMB status in patients with BLCA. The results revealed an intrinsic connection between radiomic features extracted from pelvic CECT and TMB status. Besides, we established a radiomic features-based model for TMB-prediction. Our work proved the feasibility of using radiomics to predict TMB. Yet, future study with a larger cohort is needed to verify our findings.

## Supplementary Information

**Additional file 1: Table S1.** Baseline factors between those of clustering group A and B.

**Additional file 2: Fig. S1.** Kaplan-Meier curves showing the survival outcomes of all cases. **A.** Overall survival between patients with high and low TMB; **B.** Disease-free survival between patients with high and low TMB; **C.** Overall survival between patients of clustering group A and B; **D.** Disease-free survival between patients of clustering group A and B. TMB: Tumor mutation burden. **Fig. S2.** Correlation analysis among radiomic features sequentially selected by univariate Logistic regression (**A**), LASSO regression (**B**) and backward elimination regression (**C**). Each value represents the correlation coefficient between two radiomic features.

## Data Availability

The data analyzed in this study are openly available and can be found here: http://www.cancerimagingarchive.net/ (TCIA database); http://cancergenome.nih.gov (TCGA database).

## Abbreviations

ICIs: Immune checkpoint inhibitors
PD-1: Programmed death-1
BLCA: Bladder cancer
PD-L1: Programmed death-L1
TMB: Tumor mutation burden
WES: whole-exome sequencing
CECT: Contrast-enhanced computed tomography
TCIA: The cancer imaging archive database
TCGA: The cancer genome atlas
VOI: Volume of interest
GLCM: Gray-level co-occurrence matrix
GLDM: Gray-level difference matrix
GLRLM: Gray-level run length matrix
GLSZM: Gray-level size zone
NGTDM: Neighborhood gray tone difference matrix
ICC: Interclass correlation coefficient
LASSO: Least absolute shrinkage and selection operator
ROC: Receiver operating characteristic
AUC: Area under the receiver operating characteristic curve
