# Supplementary material for: Radiomic assessment as a method for predicting tumor mutation burden (TMB) of bladder cancer patients: a feasibility study
Source: BMC Cancer. 2021 Jul 16;21:823. doi: 10.1186/s12885-021-08569-y (PMC8285848; doi:10.1186/s12885-021-08569-y)
Supplement: Supplementary file 1 — Additional file 1: Table S1. Baseline factors between those of clustering group A and B. [file 12885_2021_8569_MOESM1_ESM.docx]

**Table S1. Baseline factors between those of clustering group A and B.**

|  | **Total**  **N=75** | **Clustering group A**  **(N=24)** | **Clustering group B**  **(N=51)** | ***P***  **value** |
| --- | --- | --- | --- | --- |
| **Age (Y)** |  |  |  |  |
| **<69** | 38(50.7%) | 9(37.5%) | 29(56.9%) | 0.118 |
| **≥69** | 37(49.3%) | 15(62.5%) | 22(43.1%) |  |
| **Gender** |  |  |  |  |
| **Male** | 57(76.0%) | 17(70.8%) | 40(78.4%) | 0.472 |
| **Female** | 18(24.0%) | 7(29.2%) | 11(21.6%) |  |
| **Race** |  |  |  |  |
| **White** | 67(89.3%) | 22(91.7%) | 45(88.2%) | 0.767 |
| **Black** | 7(9.3%) | 2(8.3%) | 5(9.8%) |  |
| **Asian** | 1(1.3%) | 0(0.0%) | 1(2.0%) |  |
| **Diagnosis Year** |  |  |  |  |
| **2005-2010** | 30(40.0%) | 11(45.8%) | 19(37.3%) | 0.479 |
| **2011-2013** | 45(60.0%) | 13(54.2%) | 32(62.7%) |  |
| **BMI (kg/m2)** |  |  |  |  |
| **<26.6** | 34(45.3%) | 13(54.2%) | 21(41.2%) | 0.265 |
| **≥26.6** | 35(46.7%) | 9(37.5%) | 26(51.0%) |  |
| **Unknown** | 6(8.0%) | 2(8.3%) | 4(7.8%) |  |
| **p T stage** |  |  |  |  |
| **pT2** | 24(32.0%) | 12(50.0%) | 12(23.5%) | 0.014 |
| **pT3-4** | 43(57.3%) | 9(37.5%) | 34(66.7%) |  |
| **Unknown** | 8(10.7%) | 3(12.5%) | 5(9.8%) |  |
| **p N stage** |  |  |  |  |
| **pN0** | 42(56.0%) | 11(45.8%) | 31(60.8%) | 0.838 |
| **pN1-2** | 21(28.0%) | 5(20.8%) | 16(31.4%) |  |
| **Unknown** | 12(16.0%) | 8(33.3%) | 4(7.8%) |  |
| **Stage** |  |  |  |  |
| **Stage Ⅱ** | 28(37.3%) | 13(54.2%) | 15(29.4%) | 0.039 |
| **Stage Ⅲ** | 47(62.7%) | 11(45.8%) | 36(70.6%) |  |
| **TMB** |  |  |  |  |
| **Low TMB** | 38(50.7%) | 8(33.3%) | 30(58.8%) | 0.039 |
| **High TMB** | 37(49.3%) | 16(66.7%) | 21(41.2%) |  |

TMB: Tumor mutation burden; BMI: Body mass index
