# Supplementary material for: Radiomic assessment as a method for predicting tumor mutation burden (TMB) of bladder cancer patients: a feasibility study
Source: BMC Cancer. 2021 Jul 16;21:823. doi: 10.1186/s12885-021-08569-y (PMC8285848; doi:10.1186/s12885-021-08569-y)
Supplement: Supplementary file 2 — Additional file 2: Fig. S1. Kaplan-Meier curves showing the survival outcomes of all cases. A. Overall survival between patients with high and low TMB; B. Disease-free survival between patients with high and low TMB; C. Overall survival between patients of clustering group A and B; D. Disease-free survival between patients of clustering group A and B. TMB: Tumor mutation burden. Fig. S2. Correlation analysis among radiomic features sequentially selected by univariate Logistic regression (A), LASSO regression (B) and backward elimination regression (C). Each value represents the correlation coefficient between two radiomic features. [file 12885_2021_8569_MOESM2_ESM.docx]

**Figure S1.** Kaplan-Meier curves showing the survival outcomes of all cases. **A.** Overall survival between patients with high and low TMB; **B.** Disease-free survival between patients with high and low TMB; **C.** Overall survival between patients of clustering group A and B; **D.** Disease-free survival between patients of clustering group A and B.

TMB: Tumor mutation burden

**Figure S2.** Correlation analysis among radiomic features sequentially selected by univariate Logistic regression (**A**), LASSO regression (**B**) and backward elimination regression (**C**). Each value represents the correlation coefficient between two radiomic features.
